# Diabetic Foot Ulcers and Epidermal Growth Factor: Revisiting the Local Delivery Route for a Successful Outcome

**DOI:** 10.1155/2017/2923759

**Published:** 2017-08-21

**Authors:** Jorge Berlanga-Acosta, José Fernández-Montequín, Calixto Valdés-Pérez, William Savigne-Gutiérrez, Yssel Mendoza-Marí, Ariana García-Ojalvo, Viviana Falcón‐Cama, Diana García del Barco-Herrera, Maday Fernández-Mayola, Héctor Pérez-Saad, Eulogio Pimentel-Vázquez, Aleida Urquiza-Rodríguez, Moshe Kulikovsky, Gerardo Guillén-Nieto

**Affiliations:** ^1^Wound Healing and Cytoprotection Research Group, Center for Genetic Engineering and Biotechnology, Ave 31 e/158 and 190, Cubanacan, Playa, P.O. Box 10600, Havana, Cuba; ^2^National Institute of Angiology and Vascular Surgery, Calzada del Cerro No. 1551, El Cerro, Havana, Cuba; ^3^Dermatology Department, Center for Surgical and Medical Research, Calle 216 y 11-B, Reparto Siboney, Playa, Havana, Cuba; ^4^Lin Medical Center, Rehov Vardia 12, 3465712 Haifa, Israel

## Abstract

Soon after epidermal growth factor (EGF) discovery, some* in vivo* models appeared demonstrating its property to enhance cutaneous wound healing. EGF was the first growth factor (GF) introduced in the clinical arena as a healing enhancer, exerting its mitogenic effects on epithelial, fibroblastoid, and endothelial cells via a tyrosine kinase membrane receptor. Compelling evidences from the 90s documented that, for EGF, locally prolonged bioavailability and hourly interaction with the receptor were necessary for a successful tissue response. Eventually, the enthusiasm on the clinical use of EGF to steer the healing process was wiped out as the topical route to deliver proteins started to be questioned. The simultaneous* in vivo* experiments, emphasizing the impact of the parenterally administered EGF on epithelial and nonepithelial organs in terms of mitogenesis and cytoprotection, rendered the theoretical fundamentals for the injectable use of EGF and shaped the hypothesis that locally infiltrating the diabetic ulcers would lead to an effective healing. Although the diabetic chronic wounds microenvironment is hostile for local GFs bioavailability, EGF local infiltration circumvented the limitations of its topical application, thus expanding its therapeutic prospect. Our clinical pharmacovigilance and basic studies attest the significance of the GF local infiltration for chronic wounds healing.

## 1. Introduction

Diabetic foot ulcers (DFUs) are a major and feared complication among the constellation of the multiorgan diabetic-associated disorders. DFU is defined as a full-thickness wound penetrating through the dermis (the deep vascular and collagenous inner layer of the skin) located below the ankle in a diabetes patient [1]. An astonishing review article by Armstrong and coworkers showed that DFU is not exclusively a limb-threatening condition. The relative 5-year mortality rate after limb amputation is 68% representing a second place only preceded by lung cancer [2]. Furthermore, ulcer recurrence is one of the most important and unsolved challenges in the current approach to diabetic foot disease [3].

The foundation of this complication resides in the inability to orchestrate a physiological multistep healing process. Diabetic subjects are prone to mount a chronification phenotype which is clinically translated in (1) failure for triggering proliferative phase/granulation tissue response, (2) meager or histologically abnormal angiogenesis, (3) impaired wound contraction, and (4) stagnant and aberrant reepithelialization process [4].

Diabetes-related peripheral neuropathy and ischemia-hypoxia owing to macro- and/or microvascular damage are major predisposing factors for DFU debut and its healing failure [5, 6]. At a molecular level, diabetic peripheral neuropathy predisposes to impaired wound healing by provoking an extensive derangement of neuropeptides that definitively controls critical events like inflammation, chemoattraction, vascular permeability, leucocytes adhesion, cytokines expression, endothelial cells proliferation, and growth factors release [7, 8]. By its side, diabetic vascular disease is associated with the most severe DFU adverse outcomes, including lower probability of healing, longer healing times, higher probability of ulcer recurrence, greater risks of amputations, and potentially higher mortality. It is well known that hyperglycemia is the primary trigger of vascular endothelial cells toxicity, which translates in vascular functional impairment, microvascular rarefaction, imperfect angiogenesis, and media and intima thickening [9, 10]. The ensuing hypoxia drives a peculiar clinical, cellular, and molecular signature that eventually aborts the healing process through a variety of deleterious mechanisms. Thus, the physiological relevance of oxygen delivery within the wound matrix is out of discussion [11]. Mounting evidences target the definitive role of epigenetic mechanisms for the onset of aberrant angiogenesis and wound chronicity in diabetes. Likewise, the apparently trivial glycemic levels and the cellular oxygen pressure are not passive bystanders; rather, they actively contribute to the cellular epigenetic blueprint reprogramming rendering a “stagnant transcriptome” [9, 12]. Accordingly, the instrumental molecular pathways routing to wound chronification seems to converge on three main cellular pillars: precocious senescence, proliferative refractoriness, and apoptosis [13, 14]. Again, we deem that epigenetic forces may drive the inability of the tissues to deal with external or intrinsic predisposing factors on the base of wound chronification and local reulcerations [15, 16].

The healing response emerges subsequently to cells exposure to alarm signals once the skin barrier is disrupted. This response is ultimately commanded by growth factors (GFs) which act as soluble messengers, establishing a communication network among the different cells populations and with the extracellular matrix. The paracrine GFs releasing sources include platelets, immune inflammatory cells, fibroblasts, endothelial cells, and keratinocytes [17].

Historical studies implicate an insufficient production and/or activity of GFs and their receptors in the diabetic wound healing failure, which would involve the immobilization of critical reparative ingredients [18–20]. Consequently, the topical administration of recombinant human GFs that dates back to almost 40 years ago arose as an encouraging alternative toward torpid healing processes. Nonetheless, the initial expectations with these “magic bullets” vanished away in about a 10-year period. To our understanding, two main factors quenched such excitement: (a) the inputs from basic science that associated GFs to malignant cells promotion and progression (for review see [21]) and (b) the setback that stemmed from clinical trials in which the topical administration of epidermal growth factor (EGF) failed to enhance the healing process of chronic wounds [22] and, unexpectedly, of acute, controlled, and experimentally induced wounds in healthy volunteers [23].

These disappointments warned about the need for additional research in GFs physiology and pharmacology as in the understanding of wound milieu biochemistry. After years of peaks and troughs, EGF and platelet-derived growth factor (PDGF) have remained as the only GFs in the clinical armamentarium for hard-to-heal diabetic wounds. The line of thought that encouraged us to fuel the hypothesis that infiltrating EGF into the lesions would lead to an effective healing was the resultant of recombining different and disperse pieces of knowledge, including those supporting the fact that topical administration is not an ideal delivery route for chronic wounds. Thus, this review manuscript is rather a reflection that links the elementary principles of diabetic chronic wounds biochemistry, with the rationale of reorienting the GFs delivery route for a successful healing outcome. Today after 15 years of experience, the basic science has been validated by the clinical routine.

The literature search was based on key words introduced in PubMed and Bioline International (http://www.bioline.org.br/) data sources, while only articles in English language were downloaded.

## 2. The Diabetic Wound Environment

As elegantly reviewed by Keating and El-Osta, in a broad conceptual scenario high glucose concentrations may modify gene transcription in vascular and inflammatory cells. Therefore, glycemic levels can drive important epigenetic changes imposing a continued activation of the nuclear factor-kappaB (NF-*κ*B)-p65 and downstream inflammatory promoters. From “the hard-to-forget” hyperglycemic experience, diabetic individuals will have to somewhat afford a perpetual deregulation on the inflammatory homeostasis and superoxide metabolism pathways [24]. It is very well established that a state of low grade inflammation seems to precede and even predict the clinical onset of diabetes (for review see [25, 26]). Among other consequences, this “systemic inflammation” disrupts the insulin receptor-mediated anabolism via NF-*κ*B and c-Jun N-terminal kinases systems pathways activation [27–29].

Within the context of the DFU, the cells and the proinflammatory cytokines are the same as for nondiabetic wounds; however, in diabetics, inflammation is more a condition than a transient reaction. Diabetes predisposes to inflammation, which is also driven by impairment in the mechanisms of its resolution within the healing process. Thus, we describe diabetic chronic wounds as a proinflammatory organ superimposed into a metabolically deregulated host. It is likely that proinflammatory cytokines, such as tumor necrosis factor *α* (TNF-*α*), interleukin-6, and interleukin-1*β*, produced by the wound-homed inflammatory cells, enrich their pool in the central circulation [4].

Macrophages play an instrumental role in tissues homeostasis, inflammation, and repair. It is interesting indeed that macrophages' energetic metabolism appears to precondition its functional phenotype. For the diabetic context, this notion is obviously relevant as the comparison of classically activated “inflammatory” macrophages (CAMs) and alternatively activated “reparative” macrophages (AAMs) shows definitive metabolic differences. In general, CAMs are highly dependent on aerobic glycolysis, whereas AAMs utilize fatty acid metabolism and mitochondrial oxidative phosphorylation (for review see [30]). Proinflammatory M1 macrophage subclass which is an indicator of chronic inflammation is increased in the skin of diabetic laboratory animals in comparison with nondiabetic counterparts. Conversely, the anti-inflammatory/regenerative M2 subclass is in deficit [31–33]. In line with this, since inflammatory signals suppress tricarboxylic acid cycle flux, we can hypothesize the existence of an interconnected loop in which inflammation contributes to M1 polarization, whereas this proinflammatory macrophages subclass further overhauls the local inflammatory condition. Continuing with this notion, it has been revealed that macrophages from diabetic wounds suffer from impairment in dead cell clearance activity (efferocytosis) which hinders inflammation resolution. At the wound site, efficient dead cell clearance is a prerequisite for the timely control of inflammation and a successful progression toward the proliferative phase [32].

This deleterious inflammatory impact is further accentuated by its pathogenic association with the oxidative stress and the free radicals spillover, thus generating a relentless state of multicellular cytotoxic insult. Furthermore, inflammation and infection are episodes mechanistically linked to the orchestration of a procatabolic, prodegradative phenotype within the wound milieu [34]. Again, NF-*κ*B, a well-known proinflammatory and a redox sensitive transcription factor, seems to be crucial in the activation of many inflammatory and “response-to-injury” genes, including those coding for matrix metalloproteinases (MMPs). Consequently, the deregulation of the required balance of MMPs will sharply limit the process of granulation tissue formation and maturation.

The regulatory role of cutaneous neuropeptides in controlling granulation tissue growth, maturation, and reepithelialization is of paramount significance for a physiologic healing trajectory. Contrariwise and as mentioned above, diabetic wound environment is also characterized by an imbalanced tuning between local neuropeptides, proinflammatory cytokines, and the downstream proliferative response [8, 35, 36]. Illustratively, substance P and calcitonin gene-related peptide (CGRP) neuropeptides that appear downregulated in diabetic wounds play a major role in the modulation of inflammatory and angiogenesis phases by modulating a pool of cytokines and growth factors as vascular endothelial growth factor (VEGF) and transforming growth factor-*β*1 (TGF-*β*1) [37].

TGF-*β*1 stands as a master regulator of the wound healing processes by controlling inflammation, promoting fibroblast proliferation and angiogenesis, stimulating collagen synthesis, and favoring the deposition and remodeling of the new extracellular matrix. Its deficit or a failure with the receptors axis is definitively associated with chronic nonhealing wounds [38]. TGF-*β*1 biological activity, and therefore indirectly the healing trajectory and ultimately its esthetic outcome, is counterbalanced by TNF-*α*/NF-*κ*B in macrophages and fibroblasts [39]. Experimental evidences and mathematical models have led to the conclusion that any therapeutic approach aimed at neutralizing TNF-*α* or increasing the local availability of active TGF-*β*1 would be similarly effective in enhancing DFU healing [40].

Germane for the cytotoxicity within the diabetic wounds environment and in close pathogenic partnership with the inflammatory effectors are the disproportionate generation of reactive oxygen species (ROS) and the accumulation of advanced glycoxidation-end products (AGEs). Hyperglycemia-originated and mitochondrial-related superoxide radicals create a prooxidative atmosphere that impairs wound healing. ROS indistinctively induce the onset of both senescent and apoptogenic programs in fibroblast, keratinocytes, and endothelial cells, dismantling the wound healing process [41, 42].

AGEs are perhaps one of the most pathogenically significant contributors to diabetic complications. This family of chemicals elicits the nonenzymatic modification of proteins including the cutaneous elastin and collagen. Glycation-derived free radicals can also cause protein fragmentation and oxidation of nucleic acids and lipids. All these toxic chemical modifications per se induce premature ageing of the skin cells in diabetic individuals [43]. Of paramount pathogenic significance is the recent evidence that shows that AGEs could be involved in the process of autophagy in multiple cell types, including the skin fibroblasts. It is likely that a diabetes-mediated excessive threshold of autophagy could lead to fibroblasts function impairment, depopulation, and ultimately to a nonhealing chronic phenotype [44].

Interaction of AGEs with their cellular receptors (RAGE) ensures their important role in the pathogenesis of all the diabetic complications. RAGE acts as a signal transduction receptor for Nɛ-(carboxymethyl lysine), the major AGE* in vivo* [45]. This receptor's expression is enhanced along diabetes progression and its occupation by AGEs appears as a keystone event for the development and progression of diabetic complications. RAGE occupation causes oxidative stress and activation of NF-*κ*B via the p21ras and the mitogen-activated protein kinase signaling pathway. In the aftermath, more proinflammatory cytokines are produced, adhesion molecules are overexpressed, and ROS generation is amplified. Definitely diabetes complications are driven by multiple but redundant and concatenated inflammatory, oxidative, and toxic constituents [46].

## 3. Chronology of Topical Administration Failures: The Basic Science Is Ignored

To the best of our knowledge the first clinical disappointment for EGF treatment in the context of chronic wounds emerged from the work of Falanga and coworkers, when they approached speeding up the closure of venous ulcers following applying EGF in a liquid formulation [22]. Diverging from the inaugural achievement of the first clinical intervention with topically administered EGF in a semisolid cream for donor sites in thermally injured patients, as a model of acute wound [47], few years later, Cohen and coworkers published the broadly cited article in which topically administered EGF in a semisolid cream failed to enhance reepithelialization in healthy volunteers with controlled partial thickness wounds [23]. A critical and comprehensive analysis conducted by Falanga [48] suggested the prerequisite to gain further understanding on the biological basis of chronic wound cells and the local milieu biochemistry. Thereafter, myriad of investigations plagued the literature supporting the need of intervening to modify wound local factors as to ensure an appropriate GFs pharmacodynamic response. Others claimed the need of GFs combinations as the optimal tool to restore the healing trajectory in chronic wounds [49].

The irruption of GFs in the clinical arena appeared premature in relation to the basic science supporting its molecular pharmacology in the context of complex and chronic wounds [50]. This statement is likely based on the classic observation published by Davidson's group in 1985 [51] in which they demonstrated that EGF wound healing enhancement in mice was overtly promoted under the form of a prolonged, sustained, slow release system. This formulation translated in a dramatic fibroblasts proliferation, secretion, and matrix organization. This revolutionary Davidson et al.'s innovation assimilated the classic concept that EGF required a constant exposure to its receptor. It means that the EGF-related wound healing properties could happen if the receptors are exposed by at least 8 to 12 hours when the receptors are steadily occupied and a mitogenic signal is eventually transduced [52–54]. These enlightening concepts of basic science did not appear to be broadly embraced by formulations designers and clinicians.

**Figure 1:**
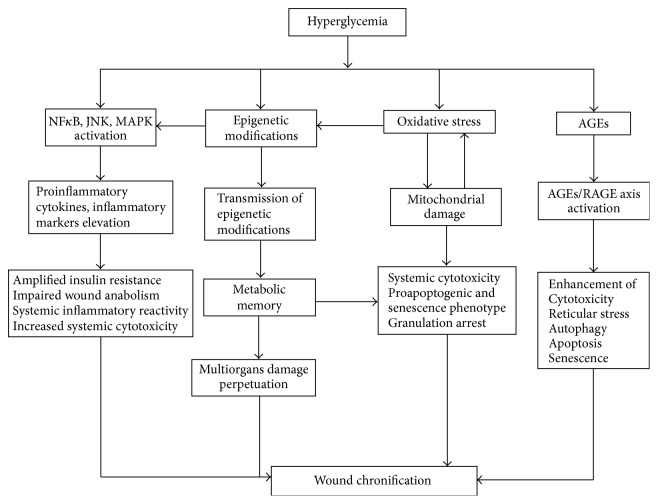
Impact of high glucose burden in multiple organs and tissues complications. Cells exposure to high glucose concentrations is harmful. Hyperglycemia triggers the activation of transcription factors that impose a proinflammatory phenotype which may also increase circulating levels of proinflammatory cytokines. This inflammatory/reactive condition further amplifies insulin resistance and raises the accumulation of more inflammatory cells within the wound. By its side, insulin resistance hinders the proanabolic function of insulin. Inflammation perpetuation and anabolism breakdown contribute to imposing a poorly synthetic, prodegradative environment in the wound. In fact, some of the inflammation-activating transcription factors are also involved in matrix proteases transcription activation. The role of epigenetics is increasingly filling gaps and has explained the molecular bases of the metabolic memory. Oxidative stress by an excessive and uncontrolled generation of free radicals is a sine qua non condition of diabetes. Pivotal roles are primarily played by free radicals in damaging mitochondrial structures which further ensures more radical toxicity. The damage spectrum includes reticular stress and apoptosis, autophagy, growth factors receptors signaling disruption, orchestration of a precocious senescence program, and proliferative arrest. All these factors disrupt the healing cascade and contribute to wound chronification. Similar toxicity is generated by the accumulation of the advanced glycation or glycoxidation-end products (AGEs). AGEs contribute to wound chronification by multiple roads that include deterioration of the innate immune mechanism with the ensued infection and perpetuation of procatabolic and proinflammatory conditions, so as to induce fibroblasts and endothelial cells apoptosis.

Conclusively diabetic chronic wounds microenvironment is hostile for local GFs stability, chemical integrity, bioavailability, and ultimately to their physiological role as major drivers for the healing process (Figure 1). Within this environment the receptors steady expression and signaling ability are impaired. These evidences lead us to suggest that diabetic wound cells are embedded in a GFs-mediated tyrosine kinase negative balance, which would presuppose proliferative arrest, and reduced cytoprotective reserves and a precocious senescent phenotype [55–59].

## 4. Major Inconveniences along the Topical Administration of EGF and Other GFs

In an attempt to enhance the healing process of a variety of peripheral wounds, EGF topical administration was inaugurated in 1989 (16 clinical reports). This growth factor has also been orally and rectally administered for gastrointestinal damage (11 clinical reports), while exhibiting therapeutic efficacy and excellent tolerability. Lack of long-term adverse effects is highlighted in different studies with 6, 12, and 24 months of patients' follow-up [60]. However, and in sharp contrast with the concomitant success achieved with the parenteral administration of EGF for premature neonates with evidence of necrotizing enterocolitis [61], its pharmacodynamic response appeared frustrating when it was topically administered to treat acute and chronic cutaneous wounds [60, 62].

During the early 90s we accrued the experience of injecting EGF locally into rats' hind limbs denervated upon sciatic nerve full-thickness cut. In addition to significantly assisting in neurological restoration, the treatment enhanced limbs peripheral soft tissues survival by delaying or preventing the onset of plantar ulcers and toes necrosis [63]. These experiments offered an important lesson: locally injected EGF could stimulate the survival and repair of cutaneous and adjacent soft tissues in a context of circulatory neurogenic deterioration. Trophic ulcers appeared to be prevented. We subsequently showed in a variety of pathological models that single or repeated EGF systemic or local injections exerted “clear-cut” cytoprotective and proliferative responses supporting the intrinsic ability of EGF at supraphysiological concentrations to unleash biological events required for tissue repair [21, 64].

These pieces of knowledge contributed to shaping the idea that injecting EGF deep into the wound base and contours would allow for a larger pharmacodynamic response in terms of granulation tissue growth and wound closure. In a compassionate study with terminal ulcer patients in 2001-2002, the first clinical evidence using EGF infiltration for diabetic foot ulcers and amputation residual bases emerged [65]. All lesions were chronic, complex, and recalcitrant, Wagner scale stages 3 and 4. Efficacy demonstrated in these wounds paved the way for solid clinical development, which culminated in a nationwide, double-blind, placebo-controlled phase III clinical trial, duly registered with our national regulatory agency and the insertion of this type of intervention within the integral program for diabetic foot ulcers care [66]. Further nationwide pharmacovigilance studies data have confirmed that the clinical performance of this procedure fits with the clinical trials results, both in terms of safety and efficacy, with 75% probability of complete granulation response, 61% of complete healing, and a 16% absolute and 71% relative reduction of the risk of amputation. Furthermore, recurrences are reported as an exceptional event upon a 12-month follow-up period [67, 68]. Other international groups who have introduced this delivery route have converged in reporting that EGF intralesional application after infection control provides high healing response with low amputation rates [69–71].

Herein, we enlist some inconveniences associated with EGF and other peptide growth factors' topical administration.

**Figure 2:**
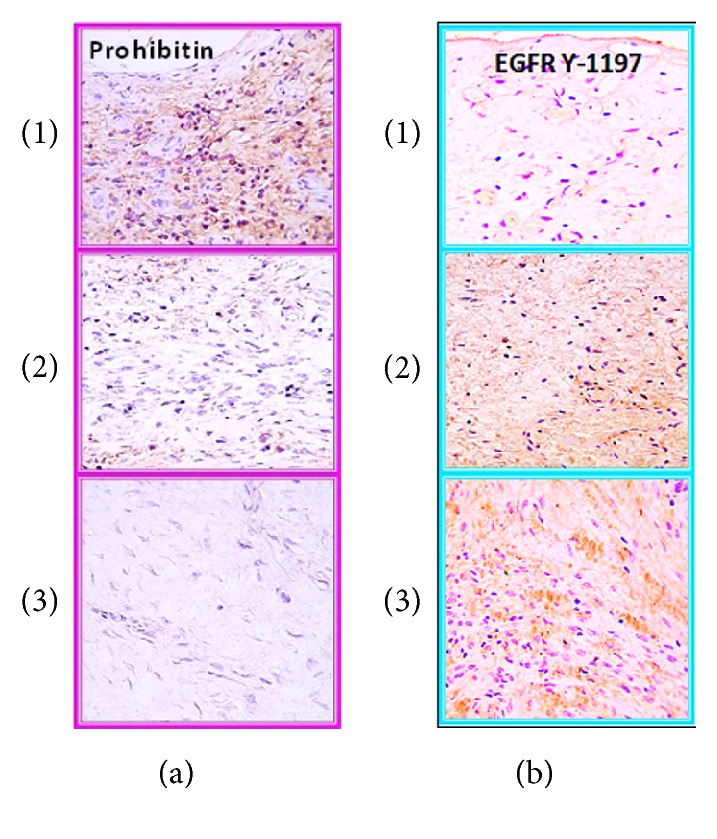
EGF receptor (EGFR) and prohibitin expression along the longitudinal axis of granulation tissue collected from neuropathic diabetic foot ulcer. Three strata ((1)–(3), where (1) is the wound surface) along the longitudinal axis of the biopsy material (approximately 2 mm/strata and 6 mm depth) were clearly distinguished according to the type of cellularity and the spreading, intensity, and definition of the immunolabelling. (a) Prohibitin expression. Prohibitin is a well-characterized protein involved in cell cycle arrest. As shown, the peroxidase-derived brownish label is far more concentrated on the wound surface (layer (1)). (b) EGFR expression. Recognition of EGFR phosphorylated on tyrosine residue 1197 indicates downstream signaling activation, prevailing in layer (3). As noticeable, EGFR expression is absent from the wound surface layer. Thus, an inverse expression pattern is shown for both markers which suggests the differential biological response of the cells of each layer. A representative aspect of each stratum was photographed and composed as in the slide. Summing up, EGFR immunostaining prevails at the wound bottom and not in its surface. In contrast prohibitin appears far more expressed on the wound surface. Pictures were obtained at ×40 constant magnification.

- *Wound Bed Preconditioning*. Standard debridement (edge of wound), moist and infection/inflammation controls, and in general the concept of wound bed preparation (known by the acronym of TIME) was first developed about 14 years ago, as an attempt to provide a framework for a structured approach to transform the chronic wound substrate into an optimized groundwork [72]. Obviously this relatively new, revolutionary, and holistic approach assumed and routinely practiced today had not been coined at the early times when the GFs were introduced in the clinical arena. This could explain the controversies on the efficacy of topically administered GFs as demonstrated by the winding and slippery slope of Regranex development in the USA. According to Smiell et al.'s analysis of four randomized studies, combining surgical debridement with the daily topical application of PDGF-BB, a modest 15% improvement in the rate of fully healed diabetic ulcers was showed as compared to placebo [73]. Despite the progresses achieved in the basic understanding and the clinical management of chronic wounds, the use of GF doses and administration regimens remain as empiric as 30 years ago, which is likely related to the lack of broad, prospective, dose-controlled clinical trials.
- *Limited Diffusion of Topically Administered GFs*. One of the greatest advances in this GFs story was offered by Cross and Roberts in 1999 [74]. Basic fibroblast growth factor and epidermal growth factor only penetrated slightly into the upper granulating layers of the wound site, showing an exponential decline in solute concentration with tissue depth for all solutes in the wound and underlying area. This absorption kinetic calculation-based assumption explains why topically administered GF was showed to fail in the clinical arena. Diffusion limitations into the wound bed deep layers appeared to be critical irrespective to the phase of the healing process.
- *Lack of Pharmacodynamic Understanding*. The amount, or pharmacologically speaking, the dose of a given GF appears to change along the different phases of the healing process, which is dictated by the maturation of the wound matrix and its cellularity. Nevertheless, the appropriate GFs doses, their biologically justified combinations, and the appropriate opportunity therapeutic window still remain elusive [74].
- *Nonreceptive Granulation Tissue Superficial Cells*. In a continuum of this illuminating work we demonstrated via immunohistochemistry with specific antibodies that, in superficially sharp debrided neuropathic ulcers, the EGF receptor (EGFR) is not located on the wound surface cells layer but in deeper strata of fibroblasts. This is particularly relevant for the catalytic domain of tyr-1197 residue, which is involved in cell survival, motility, and proliferation. On the contrary, the wound surface cells are far more abundant on the expression of prohibitin a cell cycle arrest protein. These findings suggest that topical administration pharmacodynamics is questionable by two major issues or limiting factors: limited diffusion to lower wound layers and the lack of EGFR on the wound surface cells; conversely they are plagued with an antimitogenic protein (Figure 2).
- *Local GFs Degradation.* A relevant ingredient for topically applied growth factor pharmacology is bioavailability and local pharmacokinetic. Rigorous experiments evidences since early 90s from Cohen and Schultz laboratories demonstrated the activity of locally secreted proteases in chronic wounds against GFs and their locally expressed receptors. Chronic wounds exudate has proved to degrade a myriad of natural and synthetic substrates and derive from inflammatory cells, lytic cells, and local planktonic bacteria [75, 76]. Conversely, this degradative hostile microenvironment was ameliorated with the inclusion of protease inhibitors. The addition of these antiproteolytic agents proved to enhance and speed up the closure of controlled burn wounds exposed to EGF, highlighting the need to locally preserve the GF. Furthermore, with protease inhibitors locally administered onto the wounds, EGF proved to exhibit broad systemic effects in terms of anabolism [77–79]. Furthermore, the diabetic ulcer biofilm is made up by a group of genotypically distinct bacteria that symbiotically produce a polymicrobial community. The understanding of the pathogenic significance of the biofilm in chronic wounds rendered explanation on why topically administered GFs may have failed in healing some of these lesions [80, 81]. Our group also showed that a clean, sterile exudate, obtained from full-thickness controlled acute wound in Yorkshire pigs maintained under laboratory conditions, exhibits a potent proteolytic effect on a synthetic fluorescent substrate with an amino acid sequence similar to an EGF molecule fragment [82].
- *Prolonged Interaction with the Cell Receptors*. We also determined that ^125^I-EGF formulated with a semisolid vehicle was rapidly cleared from the application site, probably by protease-driven cleavage and receptor-mediated endocytosis. Mean residence time values suggested that over 60% of the amount administered could have disappeared as early as two hours after administration [83]. These Prats and coworkers' evidences appear to reinforce previous paradigmatic findings which provided the elementary principles for an EGF-mediated cellular mitogenic response [51, 53, 54].

All this briefly summarized history elicited new strategies aimed at reformulating the topically administered EGF or redesigning its delivery route. This became mandatory, together with the wound bed preparation, as to ensure local bioavailability principles and full EGFR tyrosine kinase activation.

## 5. Major Factors Supporting the Evidences of the Infiltrative Administration

In a brief manner, injecting EGF down into the base and contours of the wounds, including the dermoepidermal junction, appears to (1) reduce its local degradation, (2) jump over the diffusion limiting barriers, and (3) ensure its bioavailability for a prolonged interaction with the receptor, in a deep fibroblasts-populated stratum along the longitudinal axis of granulation tissue [84, 85].

A recent study by our group [86], in which via immunoelectron microscopy we conducted a time-point kinetic intracellular trafficking of the EGFR in ulcers-collected fibroblasts, showed that locally infiltrated EGF into Wagner's 3 and 4 neuropathic ulcers resulted in

- dramatic increase of the EGFR membrane expression 15 minutes after the EGF infiltration into the ulcer as compared to “time zero” (*T*0: prior to the intervention); this evidence suggests the induction of its own receptor by the high-affinity ligand EGF;
- immediate endocytosis of the EGFR;
- translocation and biodistribution to different cytoplasmic organelles from time of 15 minutes to 24 hours after the infiltration;
- nuclear translocation of the EGFR and DNA binding which appeared to last from minute 45 to 24 hours after the treatment;
- a concomitant activation of the proliferating cell nuclear antigen (a cell cycle promoting protein) gene transcription, since a burst of this protein was detected following EGF intervention which appeared evident even at hour 24th after the treatment;
- a significant and intriguing accumulation of the EGFR in mitochondria which peaked between the 6th and 24th hours after the infiltration;
- a significant accumulation of the EGFR bound to collagen fibers within the extracellular matrix.

It is significant for the above described findings that classic studies by Hollier et al. support the notion that EGF makes association complex with extracellular matrix proteins, thus enhancing cell proliferation and migration via a sort of natural slow deliver system [87]. All together these data suggest that EGF delivery via infiltration stimulates the EGFR in a manner that meets the classic academic paradigms, prerequisites for cell proliferation.

Another line of evidences supporting the relevance of the locally infiltrated EGF with systemic repercussion is our clinical demonstration [88] that, in a cohort of neuropathic patients, the intervention proved to significantly reduce a variety of oxidative stress markers, while it significantly restored the antioxidant reserve parameters. Furthermore, for each evaluated marker, at least 50% of patients showed favorable responses toward a reestablished redox balance. It is noteworthy that the molecular effect of EGF on redox markers was associated with a positive clinical response in terms of granulation, contraction, and reepithelialization. In this scenario, exciting is the hypothesis that EGF significantly diminished pentosidine and total AGEs serum levels in the ulcerated patients. This effect could contribute to attenuating the myriad of AGE pathway-related damage, including wound inflammation and fibroblasts apoptosis and arrest. The EGF intraulcer infiltration also tended to correct the MMP-9/TIMP-1 balance (for the latter significantly), suggesting the recovery of the equilibrium between degradative molecules and their inhibitors, which may entail the restoration of the crosstalk between extracellular matrix prodegradative and prosynthetic forces [88].

## 6. Concluding Remarks

GFs exert crucial roles during intra- and extrauterine mammals' biology. Cells migration, differentiation, and polarization, up to individuals' morphogenesis, implicates GFs commanding gradients in an exquisitely tuned time window and topographic distribution. GFs are also omnipresent ingredients of colostrum, milk, and saliva—three main fluids for mammals' adaptation, digestion, nutrition, growth, and tissue repair. These reflections highlight the evolutionary conservation and the physiological relevance of GFs. This extensive family of polypeptides is endowed with broad and multiple abilities that can impact on each and every single cell from metabolism to proliferation in health and disease. Outspoken examples exist since the 80s and 90s with overexpressing and null transgenic mice for selected GFs genes.

GFs are one of the three major pillars of regenerative medicine given its instrumental role in orchestrating tissue repair, epithelial regeneration, and cellular reprogramming for an adequate postwounding functional recovery. Given our convincement on the biological virtues of EGF, we devised its infiltrative administration to achieve the closure of option-orphans, high grade DFU. It was our hypothesis, nurtured from scattered findings, that infiltration could circumvent the limitations confronted during years of topical use. The infiltrative delivery is, therefore, the resultant of a repositioning of different pieces of knowledge that we had accrued in animal models, ex vivo and* in vitro* experiments, further enriched by valuable conclusions obtained by international researchers.

It is likely that our first experiment on the mid of the 90s in which EGF was locally infiltrated into sciatic nerve-ablated hind limbs in rats paved the way for the conception of the parenteral administration of this molecule [63], which is evolutionarily conserved from ground worms and that is able to simultaneously trigger both cytoprotective and proliferative effects. The former is prodigiously relevant for the scenario of poorly perfused ulcers. EGF is a well-reputed cytoprotective growth factor, and this activity is driven by the agonistic stimulation of the phosphatidylinositol 3-kinase (PI3K)–Akt axis by the EGFR phosphorylation [21].

Given the clinical magnitude of the wounds treated with this modality, including some with an ankle/brachial index far below 0.7, the relative risk of amputation has been reduced. As a reminder, either conventional revascularization or the endoluminal angioplasty is the only procedure to strike back the ischemic impact. These facts have positioned “the local infiltrative blockade” of the wound as a unique and first-in-class therapy. Thus, its therapeutic opportunity becomes broader as the wound approaches to be a limb-threatening entity. We deem that the EGF local infiltration has the capability within the current pharmacological armamentarium to meet this vast unmet medical need in different populations and settings worldwide.
